# A novel insertion ins(18;5)(q21.1;q31.2q35.1) in acute myeloid leukemia associated with microdeletions at 5q31.2, 5q35.1q35.2 and 18q12.3q21.1 detected by oligobased array comparative genomic hybridization

**DOI:** 10.1186/s13039-014-0063-x

**Published:** 2014-09-25

**Authors:** Eigil Kjeldsen

**Affiliations:** Department of Hematology, HemoDiagnostic Laboratory, Cancer Cytogenetics Section, Aarhus University Hospital, Tage-Hansens Gade 2, Ent. 4A, DK-8000 Aarhus C, Denmark

**Keywords:** Acute myeloid leukemia, ins(18;5), oaCGH analysis, Chromosomal insertion, Microdeletion, Cryptic chromosomal aberration, del(5q), add(18q)

## Abstract

**Background:**

Nonrandom clonal chromosomal aberrations can be detected in approximately 55% of adult patients with acute myeloid leukemia (AML). Recurrent cytogenetic abnormalities play an important role in diagnosis, classification and prognosis of AML. However, several chromosomal abnormalities have not been completely determined or characterized, primarily because of their low incidence and limited amount of data.

**Results:**

We characterized an AML patient with a novel apparently balanced insertion ins(18;5)(q21;q31.2q35.1) that was cryptic by G-banding. The rearrangement was further examined by molecular cytogenetic methods and oligobased high-resolution array CGH (oaCGH) analysis. We show that an approximately 31.8 Mb large segment from chromosome 5 bands q31.2 to q35.1 has been inserted, by a direct mechanism, into chromosome 18 between bands q12.3 and q21.1. The insertion was unbalanced with concurrent submicroscopic deletions at 5q31.2 (approximately 0.37 Mb in size), 5q35.1q35.2 (approximately 1.98 Mb in size), and 18q12.3q21.1 (approximately 2.07 Mb in size). The microdeletions affect genes on 5q and 18q that have been associated with hematological malignancy and other cancers. A novel juxtaposition of the genes *NPM1* and *HAUS1* at 5q35.1 and 18q21.1, respectively, was detected by FISH analysis. Searching the literature and the Mitelman database revealed no previously reported ins(18;5) cases. Interestingly, however, two AML patients with translocation t(5;18)(q35;q21) encompassing the 5q35 and 18q21 breakpoint regions as detected in our present ins(18;5) patient have been reported.

**Conclusions:**

It is well-known that cytogenetic abnormalities on the long arm of chromosome 5 affect hematopoiesis. However, the precise mechanism of their involvement in myeloid transformation is elusive. Our present data shed new light onto the frequent abnormalities on 5q as well as to the less frequent abnormalities observed on 18q in myeloid malignancies. In addition, we show that oaCGH analysis is a useful adjunct to revealing submicroscopic aberrations in regions of clinical importance. Reporting rare and nonrandom chromosomal abnormalities contribute to the identification of the whole spectrum of cytogenetic abnormalities in AML and their prognostic significance.

## Background

In acute myeloid leukemia (AML) recurrent nonrandom chromosomal aberrations occur in approximately 55% of the patients. Until now about one hundred different chromosomal rearrangements have been uncovered in AML. The rearrangements mostly include balanced translocations, inversions, deletions, amplifications, monosomies and trisomies [1]. It is well established that cytogenetic analysis is an important prognostic factor that influences therapeutic decision-making and disease outcome because the various chromosomal rearrangements play critical roles in the molecular pathogenesis [2-4].

Myeloid malignancies are subdivided into distinct disease entities on the basis of specific cytogenetic or molecular genetic abnormalities [5]. Cytogenetic characterization defines three different risk groups: favorable, intermediate, and adverse [6]. Molecular characterization has revealed that mutations in *FLT3* and *NPM1* define molecular subgroups with prognostic relevance [7]. AML patients that do not fulfill WHO criteria for other categories are grouped together in the “AML, not otherwise specified (NOS)” category, which do not provide prognostic information. AML is a heterogeneous disease with respect to clinical and biological features. Hence, it is very important to better define less frequent chromosomal rearrangements in AML patients to identify the full spectrum of molecular prognostic factors.

Here we report the characterization of a novel cryptic insertion ins(18;5)(q21.1;q31.2q35.1) in a patient with *de novo* AML, who, as detected by oligobased high-resolution array CGH (oaCGH) analysis, also harbored three concurrent submicroscopic microdeletions 5q31.2, 5q35.1q35.2, and 18q12.3q21.1 in his leukemic cells. Two previous AML patients with the translocation t(5;18)(q35;q21), and similar breakpoints as observed in our patient, have been reported. We review these patients and discuss the possibility that the ins(18;5) detected in our present patient is a variant of this rare non-random chromosomal abnormality t(5;18).

## Case presentation

A 37-year-old male Caucasian, previously well, presented with 4–5 weeks of fatigue, increasing paleness and dyspnea. In this period and on admission there were no febrilia, infections, or signs of bleeding except for one occasion of melaena 3 weeks prior to admission. He had an unintended weight loss of five kg from 91 kg. Bone marrow (BM) examination showed marked hypercellularity with medium-sized mononuclear blasts and an 80% proportion of highly proliferative blasts, staining CD4+, CD7+, CD13+, CD43+, CD117+, CD123+, CD34-, HLA-DR+, CD56-, and TdT-. Hematological examination included a total white blood cell count of 4.49 × 10^9^/L, hemoglobin of 5.1 mmol/L and, platelets of 24 × 10^9^/L. Segmented neutrophil count was 0.70 × 10^9^/L. The patient’s father’s cousin and great grandmother in his mother’s line had leukemia. The patient had no comorbidity and had no previous history of being treated with chemotherapy or exposed to radiation. He had been smoking until 3 years prior to his AML diagnosis with an estimated pack years of 15. There was no information on possible occupational hazards.

Our patient entered the AML-17 treatment protocol (Trial reference ISRCTN55675535). This protocol is a randomized multi-arm Phase III study designed by the AML Working Group of the National Cancer Research Institute (NCRI) and the Hematology Oncology Study Group in Acute Myeloid Leukemia and high risk Myelodysplastic Syndrome (MDS) in adults (http://aml17.cardiff.ac.uk/). In this interventional treatment protocol, AML and high risk MDS patients are randomized to one of five subgroups for induction therapy, then risk assessed, and randomized to FLT3 inhibitor if mutated or high risk chemotherapy with or without mTOR inhibition. According to the protocol our patient was initially treated with DA because of intermediate-risk cytogenetics. Molecular genetic analysis of his bone marrow cells at diagnosis showed an internal tandem duplication mutation in FLT3 (*FLT3*-ITD) and *NPM1*^wt^, and was then assigned to high-risk leukemia. He received FLAG-IDA treatment according to AML-17 and obtained complete remission 28 days after admission as evaluated by pathology, flowcytometry, cytogenetics and molecular genetics.

## Results

### Cytogenetic and multicolor FISH analyses

**Figure 1:**
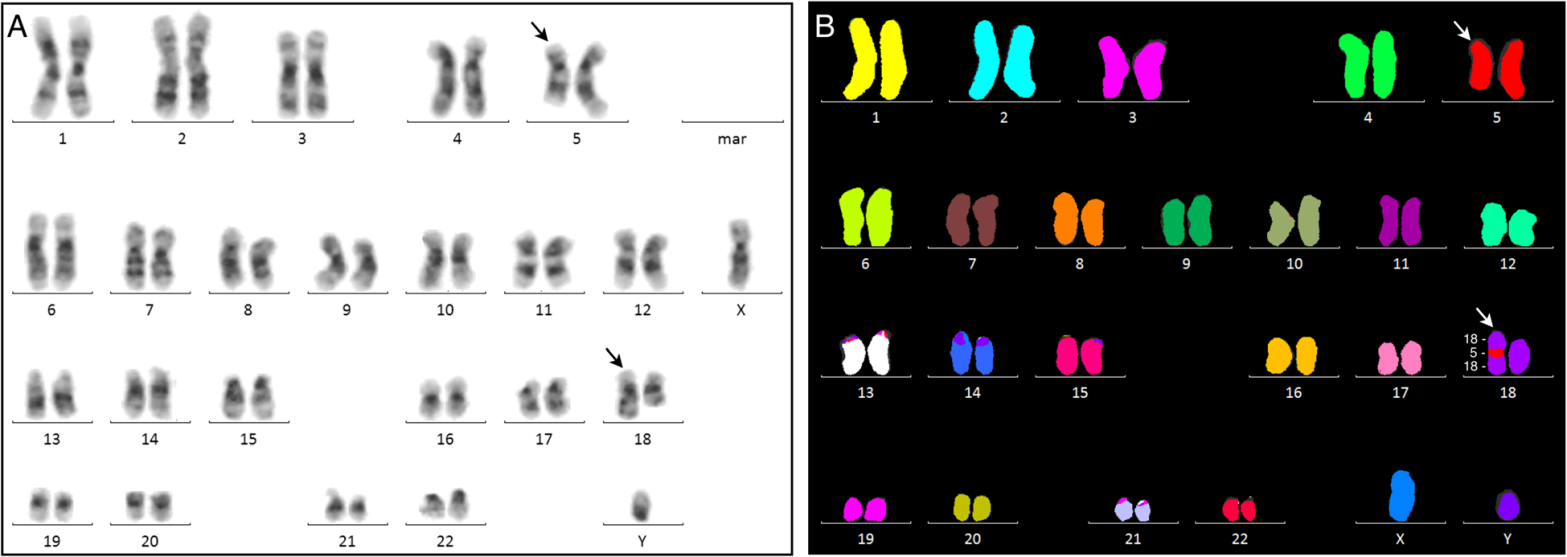
**Karyotyping analyses. A**. G-banding analysis showed an aberrant karyotype initially interpreted as 46,XY,del(5)(q22q35),add(18)(q23)[25]. **B**. 24-color karyotyping revealed the cryptic insertion ins(18;5)(q21;q31q35) in all 10 analyzed metaphases. Arrows indicate the aberrant chromosomes.

**Figure 2:**
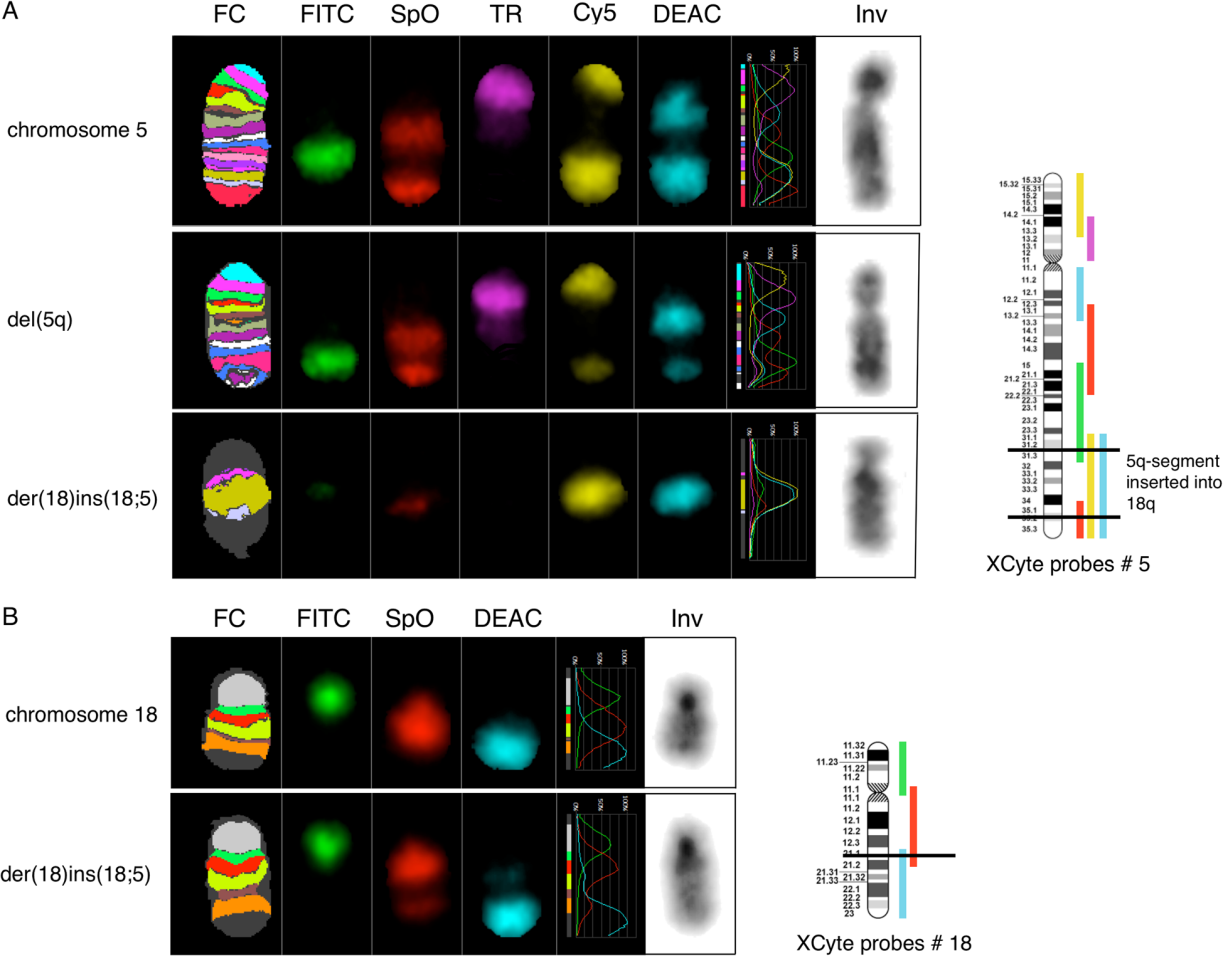
**mBanding analyses of chromosomes 5 and 18.** The single color gallery tool in the ISIS software shows assigned false colors (FC) representing individual color schemes of labeled chromosomes arranged in its capture sequence (fluorescein isothiocyanate) FITC, (spectrum orange) SpO, (texas red) TR, (cyanine 5) Cy5, (7-diethylaminocoumarin-3-carboxylic acid, succinimidyl ester) DEAC, together with an inverted gray scale image of the DAPI image (Inv). Panel **A**. mBanding analysis using the XCyte 5 probe. The top panel shows a normal chromosome 5, the middle panel shows the deleted chromosome 5, and the lower panel shows the 5q-insertion on der(18)ins(18;5). To the right is a schematic representation of the localization of the different multicolor probes of XCyte 5 relative to the ideogram of chromosome 5 together with breakpoints marked by black horizontal lines. Panel **B**. mBanding analysis using the XCyte 18 probe. The top panel shows a normal chromosome 18 and the lower panel the der(18)ins(18;5). To the right is a schematic representation of the localization of the different multicolor probes of XCyte 18 relative to the ideogram of chromosome 18 together with the breakpoint marked by a black horizontal line.

Karyotyping by G-banding of unstimulated cultured BM cells at initial diagnosis was interpreted as an apparently unbalanced male karyotype 46,XY,del(5)(q31q35),add(18)(q23)[25] (Figure 1A). To further characterize these chromosomal aberrations we next performed 24-color karyotyping using 24XCyte human multicolor FISH (mFISH) probe kit which revealed the insertion ins(18;5) and that there were no other structural abnormalities (Figure 1B). To further define the chromosome 5 segment that was cut out and where it was inserted into chromosome 18 we performed mBanding with XCyte probes for chromosomes 5 and 18 (Figure 2). These analyses showed that the chromosome segment 5q31q35 was inserted into chromosome 18 at band region q21 by a direct mechanism. By combining the obtained results a revised karyotype 46,XY,ins(18;5)(q21;q31q35)[25] could be made. Analysis of PHA-stimulated cultures of blood lymphocytes revealed a normal male karyotype, as did analysis of bone marrow cells after one induction series (data not shown). These data exclude the possibility that the observed ins(18;5) in the patient’s bone marrow cells at diagnosis was constitutional.

### oaCGH analysis

**Figure 3:**
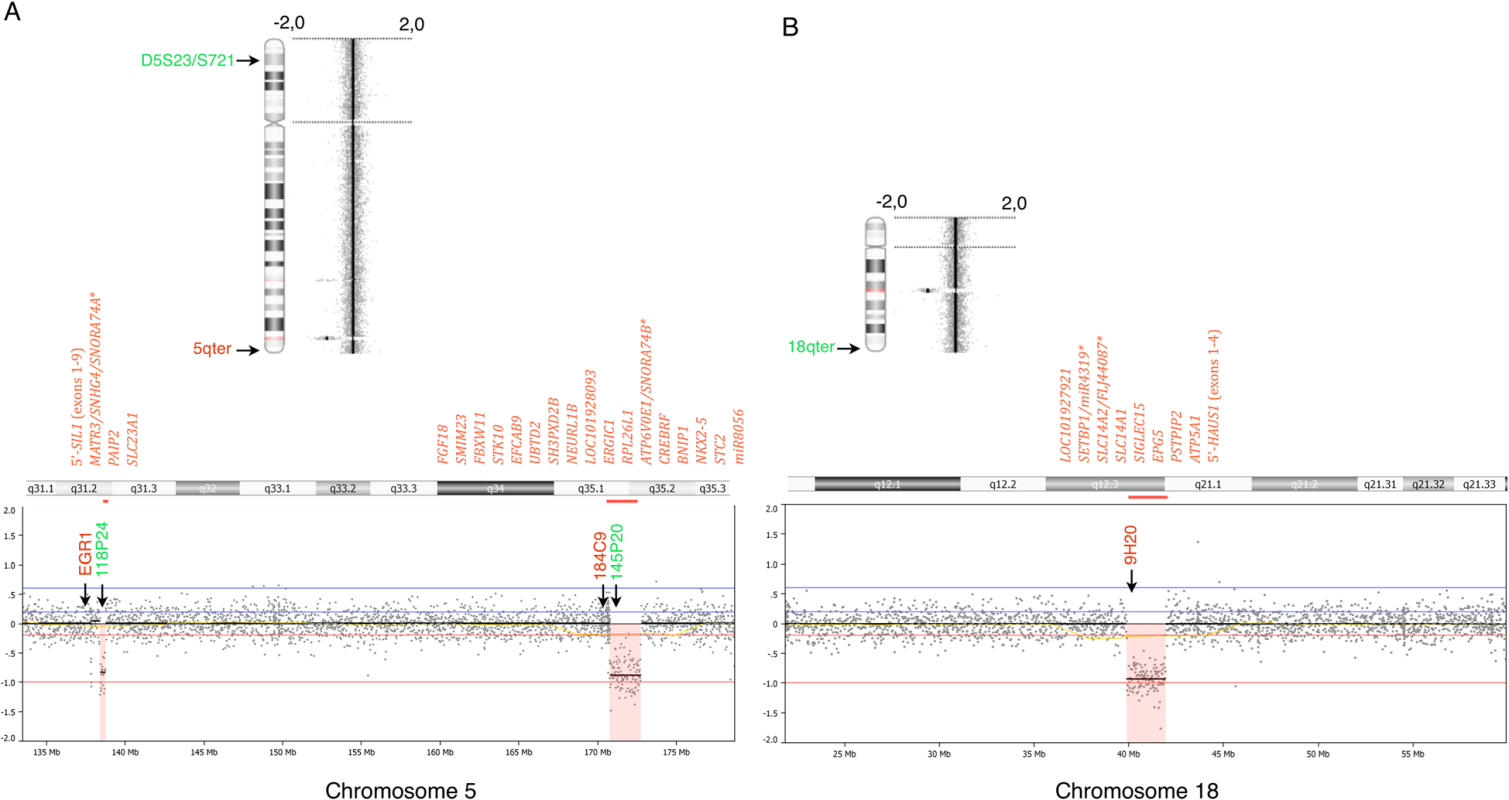
**Genome analysis using high resolution 180 K oligo-based array CGH analysis.** Panel **A**, upper panel. Chromosome 5 ideogram showing two submicroscopic deletions detected at the long arm of chromosome 5 at 5q31.2 and 5q35.1-q35.2. Lower panel, zoom view of genomic profile at chromosome 5 where the deleted regions on 5q are indicated by red shade. Panel **B**, upper panel. Chromosome 18 ideogram showing a submicroscopic deletion detected at the long arm of chromosome 18 at 18q12.3-q21.1. Lower panel, zoom view of genomic profile at chromosome 18 where the deleted region on 18q is indicated by red shade. Vertical blue lines in the zoom view indicate log_2_ ratios +0.24 and +0.60 and red lines indicate log_2_ ratios −0.24 and −1.0. The X-axis at the bottom indicates chromosomal position. The insert at the top of the genome profile indicates the chromosomal bands in the zoomed region and their relative position. The relative positions of the different FISH probes used for validation are indicated in different colors according to the direct fluorescent label used. The genes located in the minimum deleted regions are listed according to their relative genomic position (centromeric to telomeric orientation), and the asterisk (*) denotes that the genes separated by a slash represent different transcripts from the same transcriptional unit.

To search for possible copy number abnormalities involved in the ins(18;5) rearrangement we performed oaCGH analysis using the CytoChip Cancer 4×180K v2.0 (BlueGnome, Cambridge, UK). The oaCGH analysis detected four somatic copy number alterations in the form of three microdeletions at 5q31.2, 5q35.1-q35.2 and 18q12.3-q21.1 (Figure 3), and a single microamplification at 12q21.1. The microamplification had a maximal size of 158.3 kb (pos. 72,596,354-72,754,669) (Max: A_16_P19594168: 72,596,354 to A_16_P19594509: 72,754,669) and a minimum size of 107.0 kb (Min: A_16_P02650455: 72,625,008 to A_16_P19594474: 72,732,027) but this region contains no known genes (data not shown).

The maximum regions of microdeletions involved in the other break point regions are: 1) at chromosome band 5q31.2 the microdeletion encompasses the oligonucleotide probes A_16_P37384388 to A_16_P17320593 mapping from 138,390,821 to 138,769,054; 2) at chromosome bands 5q35.1-q35.2 the microdeletion encompasses the oligonucleotide probes A_16_P37464643 to A_16_P17402538 mapping from 170,768,753 to 172,758,763; and 3) at chromosome bands 18q12.3-q21.1 the microdeletion encompasses the oligonucleotide probes A_16_P41020231 to A_16_P03359511 mapping from 39,887,338 to 41,970,952. The minimum region of microdeletions in the involved break point regions are: 1) at chromosome band 5q31.2 the microdeletion encompasses the oligonucleotide probes A_16_P17319737 to A_16_P17320525 mapping from 138,403,448 to 138,746,932; 2) at chromosome bands 5q35.1-q35.2 the microdeletion encompasses the oligonucleotide probes A_18_P15493901 to A_16_P01397609 mapping from 170,771,533 to 172,741,295; and 3) at chromosome bands 18q12.2-q21.1 the microdeletion encompasses the oligonucleotide probes A_16_P20842291 to A_16_P20847704 mapping from 39,899,537 to 41,952,135. From these results the respective estimated minimum to maximum deletion sizes are: 1) at 5q31.2: 343.5-378.2 kb; 2) at 5q35.1-q35.2: 1,969.8-1,990.0 kb; and 3) 18q12.3-q21.1: 2,052.6-2,083.6 kb. The genes located in the minimal deleted regions are summarized in Figure 3.

### FISH analyses

**Figure 4:**
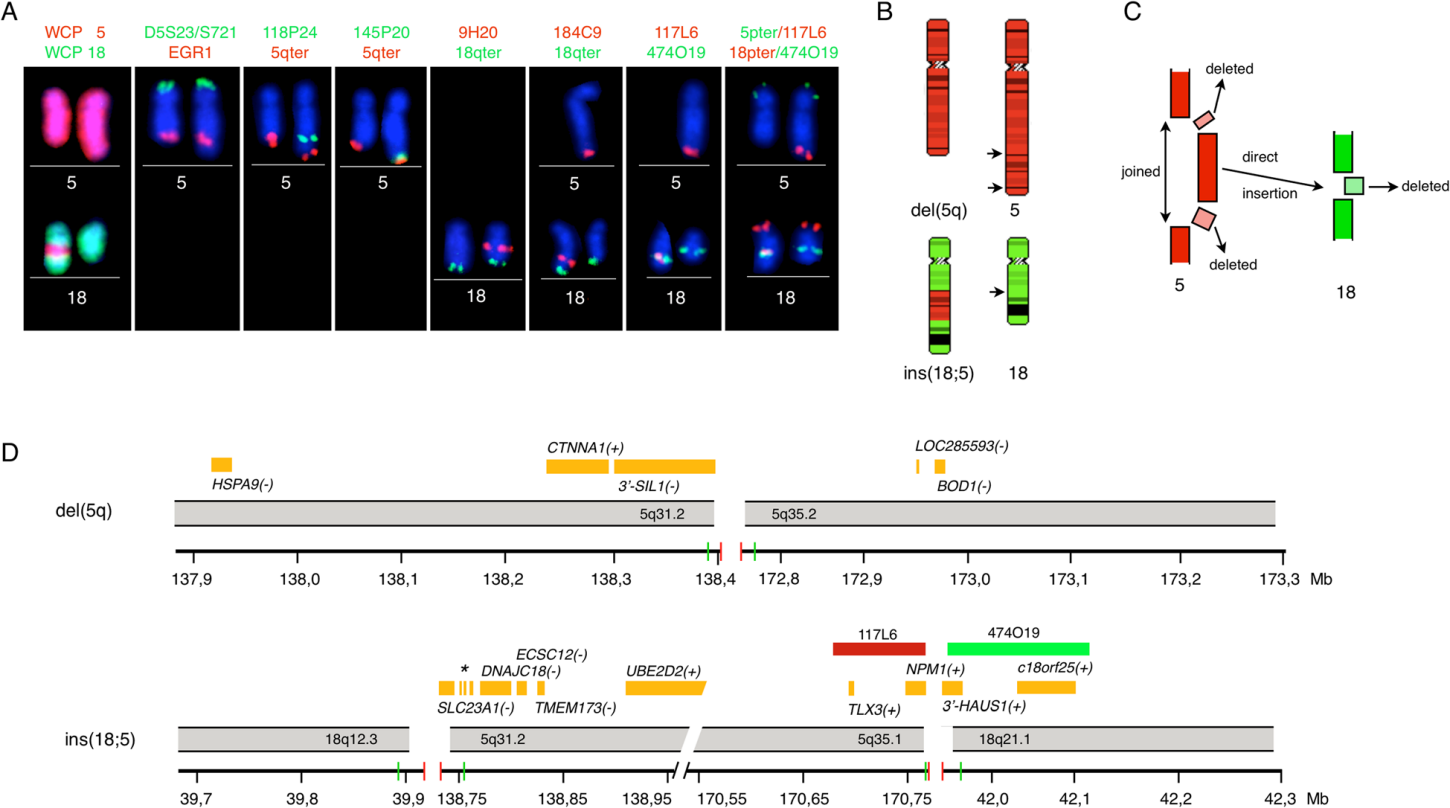
**FISH analyses for validation of array findings and a model for the generation of ins(18;5)(q21.1;q31.2q35.1).** Panel **A**. Partial karyograms of chromosome pairs 5 (upper row) and 18 (lower row) showing FISH results after hybridization using the respective probes as indicated at the top. The aberrant chromosomes are positioned to the left. The relative positions of the RP11-based BAC probes are indicated in Figure 3. For the probes RP11-117 L6 and RP11-474O19 their relative positions are indicated in Panel **D** by red and green boxes, respectively. Panel **B**. Model of the chromosomal rearrangement showing the localization of the breakpoints on the ideograms of chromosomes 5 and 18. Panel **C**. Schematic representation indicating the regions that are deleted, joined and inserted. Panel **D**. Schematic representation of genes (light brown boxes) mapping in correspondence to the breakpoint regions and each gene are indicated with respect to it genomic orientation by (+) or (−). Upper panel shows the joined region of 5q31.2 and 5q35.2 and the lower panel shows part of the directly inserted 5q31.2 to 5q35.1 fragment into 18q12.3 and 18q21.1, respectively. The deleted chromosomal fragments are omitted and the genes located in these regions are listed in Figure 3. The axis at the bottom of each panel indicates the chromosomal position of the involved regions. The resolution of the array is limited to the kilobase pair level and the density of the oligo probes differ according to chromosomal regions with the highest density at known cancer genes. Vertical red and green bars indicate the relative genomic position of deleted (red) and not deleted (green) oligonucleotide probes in oaCGH analysis. The asterisk (*) marks three minor genes in the following order *MZB1*(−), *PROB1*(−) and *SPATA24*(−).

To validate the microdeletions, FISH analyses were performed using several BAC-based custom made probes. These were co-hybridized with subtelomeric probes from 5qter and 18qter, and compared to dual color whole chromosome painting with probes for chromosomes 5 and 18 and FISH analysis with the EGR1(5q31)/D5S23,D5S721(5p15.2) dual color probe (Figure 4A). The experiments showed that: 1) the EGR1 gene is not part of the proximal microdeletion as expected from the oaCGH analysis; 2) the BAC-based probes RP11-118P24 (5q31.2), RP11-145P20 (5q35.1) and RP11-9H20 (18q12.3) all showed mono-allelic deletions confirming the microdeletions as suggested by the oaCGH analysis; and 3) the microdeletions on 5q are located on the same short derivative homologue of chromosome 5. Counting 200 interphase nuclei using each of the BAC-probes showed that approximately 90% of the interphase nuclei contained the microdeletions. Using the BAC-probe RP11-184C9 (5q35.1) it was confirmed that it is not part of the deleted region, as expected from the oaCGH analysis, but was part of the 5q fragment that was inserted onto chromosome 18. Analyzing 200 interphase nuclei with this probe a normal signal pattern of 2R2G was observed in all of the examined cells, confirming that this probe is not part of the deleted region.

Taken together we have shown that an approximately 31.8 Mb large chromosomal segment encompassing the bands 5q31.2q35.2 was cut out and inserted by a direct mechanism between chromosome bands q12.3 and q21 on chromosome 18 (Figure 4B and C). The insertion was unbalanced with concurrent submicroscopic deletions at 5q31.2 (approximately 0.37 Mb in size), 5q35.1q35.2 (approximately 1.98 Mb in size), and 18q12.3q21.1 (approximately 2.07 Mb in size). As a result of this complex rearrangement the following band regions became juxtaposed: 5q31.2-5q35.2, 18q12.3-5q31.2, and 5q35.1-18q21.1. By *in silico* analysis of the involved regions it was found that the 5′-part of *SIL1* (spanning exons 1–9) at 5q31.2 and the 5′-part of *HAUS1* (spanning exons 1–4) were deleted. A fusion of the chromosomal regions 5q35.1 and 18q21.1 spanning the *NPM1* and *HAUS1* genes, respectively, was confirmed by FISH analysis with the RP11-117L6 and RP11-474O19 probes (Figure 4D).

## Discussion

The insertion ins(18;5)(q21.1;q31.2q35.1) detected in the leukemic cells of the presented *de novo* AML patient is to the best of our knowledge a novel chromosomal abnormality. A systematic review of the literature and a search in the Mitelman database [1] did not reveal any previous reports on ins(18;5) patients with hematological or other cancers.

Insertions are very rare chromosomal abnormalities, not only in hematological malignancies but also in constitutional genetics where the incidence was estimated to be as low as 1:80,000 [8]. Despite their rarity, several instances of insertion variants of the traditional common translocations have been reported in myeloid malignancy, including ins(8;21)/ins(21;8) and t(8;21)(q22;q22)/*RUNX1*-*RUNXT1* [9], ins(3;5) and t(3;5)(q25;q35)/*NPM1-MLF1* [10], and ins(22;9) and t(9;22)(p24;q11.2)/*BCR-JAK2* [11]. Although the molecular mechanisms for generating the insertions variants are different compared to those of generating the traditional common translocations the insertion variants have similar aberrant fusion genes. Further, the AML patients with the insertion variants exhibit similar morphology and prognosis compared to their traditional translocation counterparts.

These observations prompted us to speculate whether the rearrangement observed in our patient could be a variant of a putative t(5;18). Interestingly, a revised search revealed two previously reported AML patients with translocation t(5;18)(q35;q21) [12,13] involving the same cytogenetic bands as in our patient. The clinical and genetic findings are summarized in Table 1. No additional cases could be found after a search in our cytogenetic registry containing more than 2,400 sequential entries of AML since 1990. Although all three patients share similar cytogenetic break points, the rearrangements may still be very different at the molecular level. Since there are no information regarding molecular breakpoint mapping or studies of possible concurrent submicroscopic aberrations in the reported t(5;18) patients this question cannot be addressed. From a clinical point of view it was remarkable that both patients were AML FAB subtype M2 and considered high-risk patients. For patient 2 this was because of an accompanying *FLT3*-ITD mutation while the basis for this assignment in patient 1 was cryptic. Our patient had AML with FAB subtype M1 and considered high-risk because he had an accompanying *FLT3*-ITD mutation. Patient 1 had bone marrow transplantation (BMT) in first remission. A BMT in CR1 is planned in our patient but this has been postponed because of complicating fungal infections.

**Table 1:** **Summary of published AML patients with t(5;18)(q35;q21) and present patient with ins(18;5)(q21;q31q35)**

**Reference**	**Patient 1** [12]	**Patient 2** [13]	**Present case**
**Age (yr)**	42	73	37
**Gender**	Female	Male	Male
**WBC (×10** ^**9**^ **/L)**	48.1	12.0	4.5
**Platelets (×10** ^**9**^ **/L)**	56	65	24
**Hgb (mM)**	6.0^a^	7.2^a^	5.1
**BM Morphology: Blast (%), cell size, cellularity**	80%, large size, hypocellular	60%, large size	80%, medium size, hypercellular
**Immunophenotype**	CD4+, CD13+, CD33+, HLA-DR+, CD38+, CD11c+, CD117+	CD13+, CD33+, HLA-DR+, CD117+, MPO-	CD4+, CD7+, CD13+, CD33+, CD117+, MPO+, CD56-, CD34-, TdT-
**Diagnosis**	AML-M2 (*de novo*)	AML-M2 (*de novo*)	AML-M1 (*de novo*)
**Cytogenetics**	46,XX,t(5;18)(q35;q21)[14]	46,XY,t(5;18)(q35;q21)[2]/46,XY[12]	46,XY,ins(18;5)(q21.1;q31.2q35.1)[25]
**aCGH findings**	No information	No information	0.16 Mb amplification @12q21.1
			0.37 Mb deletion @5q31.2
			1.98 Mb deletion @5q35.1q35.2
			2.07 Mb deletion @18q12.3q21.1
**Gene Mutations**	No information	*FLT3* ^ITD^	*FLT3* ^ITD^, *NMP1* ^wt^
**Outcome**	BMT in CR1	Obtained CR1	Obtained CR1, karyotype 46,XY[25],
		Relapse with t(5;18)(q35;q21) and a secondary t(3;12)(p23;p13)	BMT planned in CR1
		Died 18 months after initial diagnosis	

^a^Published values converted to SI units.

Together these observations suggest that t(5;18), and perhaps our possible variant ins(18;5), may be associated with a high risk AML *FLT3*-ITD subgroup although the contribution of each genotypic component is unknown. It is well known, however, that AML patients with a normal karyotype and a high burden of *FLT3*-ITD often present with a more aggressive disease; and more often relapse after remission [14-16]. The impact of *FLT3*-ITD among other cytogenetic subgroups is not clear [15,17].

With the aid of oaCGH analysis we detected concurrent submicroscopic deletions at each of the cytogenetic breakpoints involved in the complex rearrangement, including 5q31.2, 5q35.1q35.2 and 18q12.3q21.1. Submicroscopic deletions surrounding the most common recurrent translocations breakpoints have been reported in various leukemia with incidences ranging from approximately 2% to 20%, including t(8;21)(q22;q22), t(9;22)(q34;q11), and t(15;17)(q22;q22) [18-21]. The clinical significance of these accompanying submicroscopic deletions is largely unknown because they in some cases were associated with poor therapy response and unfavorable outcome while they in others had no effect. It was hypothesized that deletion of critical genes could account for the possible difference in disease course, or alternatively, that such deletions reflect an underlying genomic instability that may predispose the malignant cells to acquire other genetic abnormalities that confer a worse prognosis.

Simple reciprocal translocations between two non-homologous chromosomes fundamentally require only two chromosomal double stranded DNA breaks (DSBs) followed by an exchange of the resulting fragments before sealing of the breaks. A simple insertion requires three chromosomal DSBs, transfer of the segment and then sealing of the three breaks. When a simple insertion rearrangement is complicated by accompanying submicroscopic deletions in the kilobase-to-megabase size at each of the breakpoints, as described in our patient (Figure 4B and 4C), at least six DSBs are required for the complex rearrangement to occur. The additional submicroscopic deletions could either be a by-product of the chromosomal repair mechanism or part of an initiating event. The major DSB repair pathways in mammalian cells are the homologous recombination (HR) and non-homologous end-joining (NHEJ) pathways, and depending upon the chosen repair pathway aberrant chromosomal rearrangements can be generated [22]. In our patient it is likely that the error-prone NHEJ pathway repaired the respective breakpoints.

Translocations involving chromosome 5q35 are rare clonal abnormalities in hematological cancers [23]. The most common recurrent 5q35 translocations with formation of aberrant fusion genes are: t(2;5)(p23;q35)/*NPM1*-*ALK* in anaplastic large cell lymphoma [24], t(3;5)(q25;q35)/*NPM1*-*MLF1* in AML [10], t(5;17)(q35;q21)/*NPM1*-*RARA* in APL [25], in t(5;11)(q35;p15)/*NSD1*-*NUP98* in childhood AML [26], and t(5;11)(q35;q12)/*NSD1*-*FEN1* in AML-M5 [27]. The *NPM1* and *NSD1* at 5q35 are common translocation partners. The *NSD1* gene encodes a nuclear protein involved in transcriptional regulation. No prognostic mutations have been ascribed to this gene in relation to leukemia. The *NPM1* gene encodes a nuclear matrix phosphoprotein involved in nucleolar ribosome assembly and protein localization. In addition to being a translocation partner *NPM1* can be affected by mutations at the DNA sequence level. A 4 bp insertion in exon 12 in *NPM1* is one of the most frequent genetic changes known in AML patients with a normal karyotype AML; and its presence in those patients confers a better prognosis [28]. In our patient we detected no genetic abnormalities in *NPM1* except for its juxtaposition to *HAUS1* at 18q21.1. In the previously reported AML patients with t(5;18)(q35;q21) there is no information about possible aberrant fusion genes or concurrent additional abnormalities involving the *NPM1* or *NSD1* at 5q35.

Chromosomal abnormalities of the long arm of chromosome 18 are most often associated with lymphoid malignancies. However, the number of reports of genes on chromosome 18 involved in myeloid malignancy is increasing. The SETBP1 and its intronic *MIR4319* at 18q12.3 were recently described to be new players in myeloid malignancy [29,30]. *SETBP1* was shown to be overexpressed in secondary AML patients bearing the t(12;18)(p13;q12) while the intronic *MIR4319* was downregulated [30]. Although the function of *SETBP1* is unknown it has been implicated as a transcriptional regulator of many genes. Recurrent somatic mutations promote leukemic cell proliferation [29] and appear to be a poor prognostic marker especially in elderly AML patients [31]. In our AML patient we found that eleven RefSeq at 18q12.3q21.1 were completely or partially deleted (Figure 3), and that the *SETBP1* and *MIR4319* genes were two of the deleted genes. In addition, we found that the 5′-part of *HAUS1* at 18q21.1 was partially deleted and that it has become juxtaposed to *NPM1* as a result of the complex insertion-deletion rearrangement. However, with the methods we used we cannot establish whether the *NPM1* and *HAUS1* genes formed an aberrant fusion gene. *HAUS1* encodes one of eight subunits of the 390 kDa human augmin complex that is a microtubule-binding complex vital for mitotic spindle assembly [32]. There are no previous reports on chromosomal rearrangements involving this gene.

Monosomy 5 and interstitial deletions of 5q are common chromosomal abnormalities in myeloid malignancy. These aberrations occur in 5-10% of karyotypic abnormal adult AML and are usually associated with complex karyotypes, rapid disease progression and poor outcome [24,33]. In our patient we detected two interstitial microdeletions at 5q, one at 5q31.2 (between 343,5-378,2 kb in size) and another at 5q35.1-q35.2 (between 1.969,8-1.990,0 kb in size). The minor 5q31.2 deletion overlaps with the centromeric commonly deleted region (CDR) of two previously identified CDRs in myeloid malignancies [33-35]. Of the CDRs the centromeric CDR at 5q31.1-q32.2 is common in high risk MDS and in AML, while the telomeric CDR at 5q33.1 is associated with 5q-syndrom. The identification of pathogenic genes on 5q has proven to be challenging because most patients have extensive deletions encompassing both CDRs [36]. In a large SNP-based study it was found that among 1,115 examined patients with myeloid malignancies 12% had 5q deletions with a median size of 71,4 Mb ranging from 1.9 Mb to 131.28 Mb (whole arm) [37].

Since no single gene on 5q has been proven to be responsible for high risk myeloid malignancies a haploinsufficiency model has been proposed, reviewed in [33]. According to this model, loss of a single allele of more than one gene on 5q may act in concert to alter hematopoiesis, promote self-renewal of hematopoietic stem and progenitor cells (HSPCs), induce apoptosis of hematopoietic cells, and disrupt differentiation [38,39]. A number of candidate tumor-suppressor genes located at the centromeric CDR at 5q31 (including *CDC25C*, *EGR1*, *HSPA9*, *CTNNA1*, and *DIAPH1*) have been implicated in the development of high risk MDS/AML. In our patient we found that six and seventeen RefSeq genes at 5q31.2 and 5q35.1q35.2, respectively, were completely or partially deleted as illustrated in Figures 3 and 4D. It is noticed that the alpha-1 E-catenin gene, *CTNNA1,* at 5q31.2 is not involved in copy number alterations while the 5′-part of the downstream neighbor gene *SIL1* is deleted. The *CTNNA1* gene is a tumor suppressor gene that has been associated with progression and poor prognosis in leukemia [40]. The *SIL1* gene has not previously been reported to be directly involved in myeloid malignancy although it often is one of the many genes that are deleted in high risk AML/MDS patients with del(5q). It encodes a nucleotide exchange factor that is important for the function of glucose-regulated protein 78 (GRP78). GRP78 is known as a stress-inducible endoplasmic reticulum (ER) chaperone protein and serves as a master initiator of ER stress signaling [41]. Accumulation of unfolded proteins results in GRP78 activation via *SIL1* and subsequently activation of the unfolded protein response (UPR). Proteasome inhibitors, such as bortezomib, suppress the degradation of unfolded proteins and trigger ER stress leading to activation of UPR and subsequently apoptotic signals. Although bortezomib is mainly used for treatment of multiple myeloma [42] and mantle cell lymphoma [43] it has in some instances been shown to be an effective agent for treatment of 5q- MDS [44]. The combination of genes that are completely or partially deleted in myeloid malignancies with interstitial deletions of 5q might contribute to the heterogeneity of high risk AML/MDS patients.

Application of array-based CGH analysis has not only significantly improved the detection rate of chromosome aberrations in patients with hematological malignancy compared to traditional cytogenetics [45-47] but also uncovered concurrent microdeletions in patients with apparently balanced translocations [21,45]. The present study, add to the knowledge of chromosomal aberrations and indicate that oaCGH is a useful adjunct to revealing submicroscopic aberrations in genomic regions of clinical importance.

## Conclusions

The present study characterizes a high-risk *de novo* AML patient and reports on a novel rather complex insertion ins(18:15)(q21;q31.2q35.1) with concurrent submicroscopic deletions at 5q31.2, 5q35.1q35.2 and 18q12.3q21.1. The rearrangement might be a variant of the chromosomal translocation t(5;18)(q35;q21), which previously was reported in two cases with high-risk *de novo* AML. This study also highlights the clinical usefulness of oaCGH analysis to identify additional submicroscopic copy number aberrations. We have narrowed the 5q31.2 CDR in AML and provided new insight to the putative role of the 5q31.2 deletion in myeloid malignancy. In addition, we have uncovered a novel fusion of the chromosomal regions at 5q35 and 18q21.1 containing the genes *NPM1* and *HAUS1*, respectively, as a result of the complex insertion-deletion rearrangement. This study contributes to the identification of the whole spectrum of cytogenetic abnormalities in AML and their prognostic significance.

## Methods

### G-banding analysis

Chromosome analysis was done on G-banded chromosomes prepared after short-term unstimulated culturing of cells obtained from bone marrow at diagnosis, and G-banding performed on PHA-stimulated peripheral blood cells as described [48]. Karyotypes were described according to ISCN [49].

### Fluorescent in situ Hybridization (FISH) analysis

Human multicolor FISH were done according to manufacturer’s instructions using the following XCyting multicolor FISH probes: 1) 24-color karyotyping was done with the 24XCyte consisting of 24 different chromosome painting probes, 2) mBanding with XCyte 5 and XCyte 18 probes consisting of a series of partial chromosome paints for sequential partially overlapping chromosome regions of a single chromosome (MetaSystems, Altlussheim, Germany). Each of the XCyte probes was labeled with one of five fluorochromes or a unique combination thereof (combinatorial labeling). Metaphases were counterstained with 4′,6-diamidino-2-phenylindole (DAPI). Image capture was done with an automated Zeiss Axio Imager.Z2 equipped with a CCD-camera (CoolCube1) and appropriate filters using Isis software (MetaSystems). Karyotyping was done using the 24-color mFISH upgrade package, ISIS, including mBanding.

Whole chromosome painting and locus specific FISH analysis was done with the following directly labeled probes according to manufacturers’ instructions: 1) whole chromosome painting probes for chromosomes 5 and 18 (Kreatech Diagnostics, Amsterdam, The Netherlands); 2) the LSI EGR1(5q31)/D5S23,D5S721(5p15.2) dual color probe set (Abbott Molecular, Wiesbaden, Germany); and 3) subtelomeric probes for 5pter, 5qter, 18pter and 18qter (Kreatech Diagnostics). Table 2 summarizes the custom made BAC-based probes (Empire Genomics, New York, USA) for validating the oaCGH findings. Chromosomes were counterstained with DAPI. FISH results were reported according to ISCN [49].

**Table 2:** **Summary of custom made BAC-based probes for characterization and validation of oaCGH findings**

**BAC probe**	**Cytoband**	**Genomic position (bp)** ^**a**^
RP11-118P24	5q31.2	138,473,339 - 138,673,394
RP11-184C9	5q35.1	170,301,198 - 170,446,174
RP11-117L6	5q35.1	170,679,528 - 170,854,638
RP11-145P20	5q35.1	170,858,901 - 171,048,742
RP11-9H20	18q12.3	40,480,006 - 40,631,549
RP11-474O19	18q21.1	41,949,215 - 42,121,248

^a^Genomic position are given according to NCBI build 36.1 (hg18).

### Oligobased array comparative genomic hybridization analysis

oaCGH analysis was performed using CytoChip Cancer 4x180K v2.0 (BlueGnome, Cambridge, UK) encompassing a 20 kb backbone with highest concentration of probes at 670 cancer genes. The analysis was done according to manufacturer’s instructions using 0.5 μg patient DNA from bone marrow cells at initial diagnosis as described in [48]. After hybridization, washing and drying the oligo array was scanned at 2.5 μm with GenePix 4400A microarray scanner. Initial analysis and normalization was done with BlueFuseMulti v2.6. For analysis and visualization normalized log2 probe signal values were imported into Nexus Copy Number software v. 6.1 (BioDiscovery, California, USA) and segmented using FASST2 segmentation algorithm with a minimum of 3 probes/segment. Regions of gain or loss contained within copy number variable regions (CNVs) were discarded. Reference genome was NCBI build 36.1 (hg18). Bioinformatics analysis was performed by querying the UCSC database (http://genome.ucsc.edu).

## Consent

The study conforms to the provisions of the Declaration of Helsinki. Written informed consent was obtained from the patient. A copy of the written consent is available for review by the Editor-in-Chief of this journal.

## Abbreviations

BAC: Bacterial artificial chromosomes
PHA: Phytohemagglutinin
WHO: World Helath Organization
